# On-call handover ‘– if it isn't documented then it didn't happen’

**DOI:** 10.1192/bjo.2021.483

**Published:** 2021-06-18

**Authors:** Hannah Campling, Dominic Aubrey-Jones

**Affiliations:** Barnet, Enfield and Haringey Mental Health Trust

## Abstract

**Aims:**

1. To standardise the doctor handovers for on-call duties

2. To ensure there is documented evidence of handover taking place at the end of each shift

**Background:**

Since the introduction of the European working time directive the amount of hours that doctors are allowed to work has been reduced, resulting in increased handovers between teams. The National Patient Safety Committee and General Medical Council have recognised that this means we need to ensure handovers are as safe and robust as possible to ensure that patient safety is not compromised. A recent serious investigation report carried out at Chase Farm Hospital, London identified a lack of formalised handover between doctors as a contributing factor leading to patient harm. One of the recommendations of the report was for a Quality Improvement Project to be carried out in order to formalise handover.

The handover procedure at Chase Farm Hospital for core trainee doctors 'on-call' prior to this QIP was not standardised and consisted of an informal, verbal handover. Frustrations had been raised by doctors and other staff members that this current method of handover was unreliable and unsafe.

**Method:**

We sent out a questionnaire about handover to all doctors on the on-call rota to help establish what intervention would be appropriate.

We then performed a retrospective collection of documented handovers within a two month time period.

Our intervention was to introduce an email handover procedure.

Following a two month trial of this intervention, we resent the questionnaire and performed a second retrospective collection of handover documentation.

**Result:**

Prior to this QIP we found that 0% of on call handovers were being formally documented. After the introduction of our handover email 88% of handovers were being formally documented using the handover email.

Satisfaction with the handover procedure went from 0% being very satisfied and only 33% being satisfied to 50% being satisfied and 50% being very satisfied.

**Conclusion:**

A standardised and documented handover procedure is crucial for patient safety and to allow doctors to communicate jobs effectively with each other.

A secure email for handover is a successful way of formalising the handover process.

Limitations include:

Access to the handover email for new staff or locum staff.

Ensuring that doctors who aren't on the on-call rota know how to use it to handover their ward jobs.

